# Application and evaluation of the hybrid “Problem-Based Learning” model based on “Rain Classroom” in experimental courses of medical molecular biology

**DOI:** 10.3389/fmed.2024.1334919

**Published:** 2024-07-25

**Authors:** Meng Qu, Qinlong Hou, Chunyan Yu, Xushen Li, Jichen Xia, Zhiheng Dong

**Affiliations:** Department of Basic Medicine Experimental Teaching, School of Basic Medical Sciences, Beihua University, Jilin City, Jilin, China

**Keywords:** hybrid Problem-Based Learning, learning effectiveness, medical education, molecular biology, experimental courses, Rain Classroom

## Abstract

**Background:**

With the advancement of society, the cultivation of medical professionals equipped with solid theoretical knowledge, a strong sense of innovation, and critical thinking has become a crucial goal in the reform of medical higher education. Over recent years, the hybrid Problem-Based Learning (hPBL) model, a blend of Problem-Based Learning (PBL) and Lecture-Based Learning (LBL), has emerged as a novel approach in the medical education reform landscape of China. The application and efficacy of the hPBL model in medical experimental courses have piqued the interest of medical educators. The aim of this study was to appraise the application and effectiveness of the hPBL model in the experimental course of Medical Molecular Biology at Beihua University.

**Methods:**

Utilizing the “Rain Classroom” platform, students from the Preventive Medicine and Medical Imaging programs were allocated to either the hPBL or LBL method for their Medical Molecular Biology experimental courses. The hPBL model’s impact on students’ performance was evaluated across four domains: experimental theory, experimental operation, experimental report, and practical application. Questionnaires were employed to gauge students’ experiences and perceptions.

**Results:**

The results indicated that the final assessment scores of the hPBL group were significantly superior to those of the LBL group. Moreover, the hPBL model effectively amplified students’ self-learning capability, practical application skills, and communication competencies. Students expressed a high degree of satisfaction with this blended learning model.

**Conclusion:**

The hPBL model, which amalgamates PBL and LBL, has demonstrated its effectiveness in medical education. Its implementation in the experimental course of Medical Molecular Biology at Beihua University yielded positive outcomes, enhancing students’ performance and satisfaction levels. Consequently, it is recommended that the hPBL model be further promulgated in other medical experimental courses.

## Introduction

Medical molecular biology is a crucial discipline in the field of life sciences. The medical molecular biology course serves as a bridge between basic medicine and clinical medicine, as well as basic medicine and scientific research. It is also an essential foundational course for medical undergraduates in Chinese universities. Experimental courses play a vital role in the medical molecular biology curriculum, as they cultivate students’ practical skills, scientific research thinking, and innovative consciousness. However, due to its rapid development and complex, abstract content, students often exhibit low interest, resulting in suboptimal teaching outcomes. Therefore, it is necessary to explore alternative teaching methods.

Currently, Lecture-Based Learning (LBL) is widely used as the traditional teaching approach and remains a common model in basic medicine and clinical medicine (1). This method has the advantage of efficiently delivering core knowledge and concepts to learners, while allowing teachers to instruct large numbers of students. However, it has been criticized for promoting student passivity and not fully stimulating their subjective initiative in learning.

Problem-Based Learning (PBL) is a student-centered teaching approach and is considered one of the most successful educational innovations in medical education over the past 50 years (2). In PBL, students take on an active role (3, 4). This method utilizes the principles of group collaborative learning to foster students’ independent learning, problem identification, problem-solving, and critical thinking abilities through self-study, problem analysis, discussions, and group cooperation (5–8). Numerous studies have demonstrated the positive impact of PBL on medical education. Since its inception, PBL has been widely promoted and implemented in medical education institutions worldwide, including the United States, the United Kingdom, Europe, South Korea, and China (9). Several countries have even incorporated PBL courses into their national accreditation standards, such as Japan, South Korea, and China (10).

However, it is important to acknowledge that the implementation of Problem-Based Learning (PBL) can yield inconsistent effects due to cultural backgrounds, language barriers, and variations in students’ capacities across different countries and disciplines. Some critical evaluations of the teaching effectiveness of PBL have been reported (3, 11, 12). For instance, some students perceive the information conveyed through PBL teaching as ambiguous, and they find the process time-consuming and stressful (13). Choi et al. (14) reported no significant difference in learning outcomes between the PBL group and the traditional lecture-based group (14). In comparison to PBL, Lecture-Based Learning (LBL) ensures the accurate, systematic, and coherent transmission of critical knowledge to all students. Although LBL may lack in fostering critical thinking skills, previous studies have shown that some American and Swedish dental students participating in PBL studies, as well as medical students in the UK, expressed a desire for more guidance and an LBL-like teaching approach due to uncertainties about the direction and depth of knowledge learned and the excessive sense of responsibility associated with active learning (15, 16). This suggests that LBL can compensate for some of the shortcomings of PBL courses (17). A study by Yeo and Chang (18) found that approximately 78 to 93% of Korean students prefer taking PBL courses after relevant lectures (18). In contrast to other countries, in China, where there is often a large student population accompanied by a shortage of teachers, Chinese students are generally more receptive to the traditional lecture-based teaching model. The PBL teaching model is relatively new for Chinese students and greatly stimulates their interest in learning. Therefore, for Chinese students, it may be more effective to integrate the problem-oriented teaching model into the traditional teaching process, as confirmed to some extent in the study by Jia et al. (19).

In this context, a teaching model called the hybrid Problem-Based Learning (hPBL) model has been developed, examined, and implemented to combine the characteristics and advantages of both Lecture-Based Learning (LBL) and Problem-Based Learning (PBL) methods. The hPBL teaching model follows a three-stage learning approach, including pre-class pre-study, in-class discussion, and post-class review, similar to the traditional PBL model. However, the hPBL model integrates PBL discussion learning into classroom lectures, guided by the teacher.

Previous studies on the hPBL teaching model have demonstrated its effectiveness in improving students’ satisfaction and learning outcomes in various disciplines such as immunology, biology, pathology, and vascular surgery (20–24). A review conducted by Jiménez-Saiz and Rosace (17) analyzed 12 studies from different disciplines, including medicine, biology, physiology, pharmacy, and dentistry, conducted in countries such as the United States, China, Spain, Canada, India, and Turkey (17). Among these studies, 8 compared the impact of hybrid PBL models and LBL models on students’ theoretical knowledge, while 6 evaluated students’ problem-solving ability. The findings revealed that the hPBL group achieved higher theoretical scores and demonstrated better performance. Notably, one study by Carrió et al. (21) found that among undergraduates at the Faculty of Health and Life Sciences in Barcelona, the hybrid PBL approach significantly improved long-term retention of knowledge compared to the LBL approach.

In order to explore more effective teaching model for the experimental teaching of medical molecular biology among medical undergraduates, the study was designed based on the “Rain Classroom” teaching software. The “Rain Classroom” is a WeChat mini-program developed by Tsinghua University and Xuetang Online Company, known for its user-friendly interface and diverse functionalities. The “Rain Classroom” platform is available free for public use. It serves as one of the primary online teaching platforms at Beihua University. Utilizing the “Rain Classroom” platform, teachers were able to facilitate a variety of teaching activities, including online classes, sharing learning materials such as PowerPoint presentations, videos, literature, and syllabi, assigning and collecting homework, hosting after-class workshops, engaging in online discussions, and even organizing exams.

This study aimed to evaluate the effectiveness and acceptability of the hybrid Problem-Based Learning (hPBL) model in the experimental teaching of medical molecular biology, with Lecture-Based Learning (LBL) methods used as a control. The research was conducted during the fall semester of 2021 and involved undergraduate students majoring in preventive medicine and medical imaging from the class of 2019 at Beihua University in Jilin Province.

## Materials and methods

### Study participants

A total of 164 undergraduate students from the class of 2019 at Beihua University, majoring in a 5-year program of preventive medicine and medical imaging, participated in this study during their third academic year. The sex distribution of the participants was predominantly female (27.4% male, 72.6% female), with ages ranging from 19 to 22 years (mean age of 20.2 ± 1.4 years). All participants were ethnically Chinese. Admission to the university was based on the Chinese college entrance examination, ensuring a relatively homogeneous distribution of admission scores among students within the same major.

Prior to the course under investigation, all participating students completed the foundational medical curriculum in their first two academic years, encompassing subjects such as medical chemistry, cell biology, biochemistry, and medical microbiology.

The pre-course mean grade point average (GPA) was calculated for each major, which consisted of two classes: class 1 and class 2. There were no significant differences in the mean GPA between the two classes of preventive medicine (M_class1_ = 3.23 ± 0.22, M_class2_ = 3.28 ± 0.23 on a 4-point scale, *P* = 0.814) or the two classes of medical imaging (M_class1_ = 3.39 ± 0.15, M_class2_ = 3.35 ± 0.20 on a 4-point scale, *P* = 0.823).

Informed consent forms were obtained from all participants. In voluntary, without any known risk, students were randomly assigned to either the high Problem-Based Learning (hPBL) group or the Lecture-Based Learning (LBL) group, with the LBL group serving as the control group. The hPBL group consisted of 83 students, including 33 students from preventive medicine class 2 and 50 students from medical imaging class 2, with a mean GPA of 3.32 ± 0.21. The LBL group comprised 81 students, including 27 students from preventive medicine class 1 and 54 students from medical imaging class 1, with a mean GPA of 3.34 ± 0.19. There were no significant differences in the mean GPA between the hPBL group and the LBL group (*P* = 0.703).

None of the students had prior exposure to Problem-Based Learning (PBL) instruction. Under the guidance of the same group of teachers, both groups of students completed a 16-h molecular biology experimental course with identical content during the fall semester of 2021.

### Study design

**FIGURE 1:**
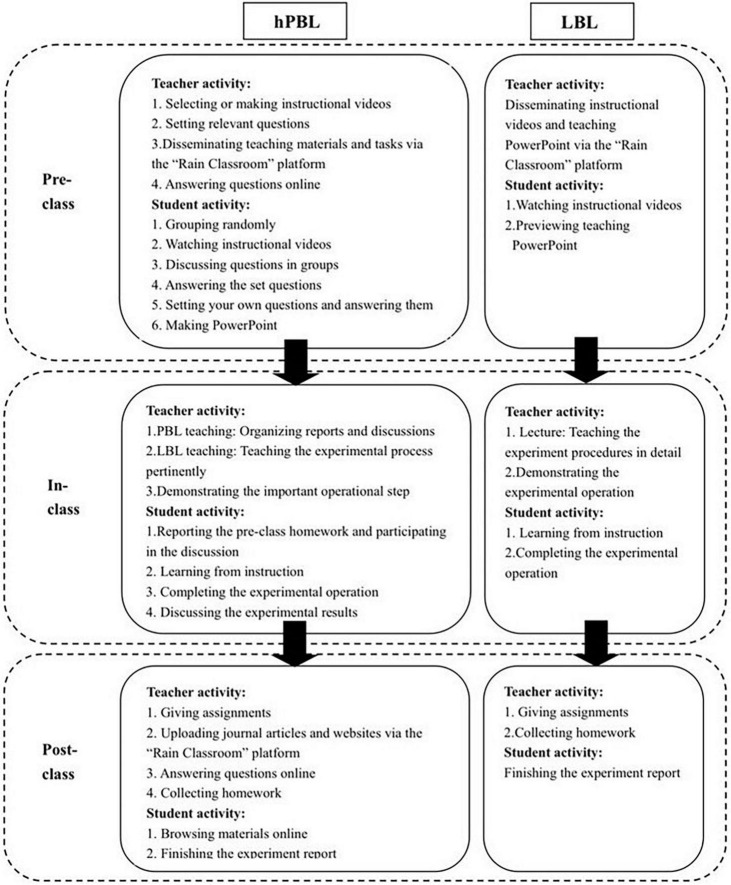
Overview of the hPBL and LBL approach design.

The primary objective of molecular biology experimental teaching is to facilitate students’ acquisition of relevant experimental skills, comprehension of experimental theory, and enhancement of their problem-solving abilities (25). To ensure comparability, feasibility protocols were designed for this study. The research principle is illustrated in Figure 1.

Despite differences in teaching dates, both the hPBL group and the LBL group received instruction in the same location, from the same instructors, and using identical lecture slides. Each session lasted 180 min, with classes held once a week for four consecutive weeks. Both groups were provided with the same textbook and course syllabus by their respective teachers the detailed information of class hour arrangement (see Supplementary Table).

The LBL groups followed a traditional experimental teaching model. One week before each class, the teacher disseminated the course content, electronic handouts, and instructional videos via the “Rain Classroom” platform. Students were encouraged to form groups for pre-class preparation, during which they could access the learning materials on the “Rain Classroom” platform. The platform recorded the students’ viewing time, providing the teacher with insights into the students’ pre-class learning engagement. In the class, the teacher commenced by explaining the experimental principles, reagents, instruments, operation methods, and safety precautions. Following the teacher’s demonstration, the students carried out the experimental procedures themselves. After the class, the students organized their experiment results, composed experiment reports, and submitted them for assessment.

In the hPBL group, students were assigned pre-class learning tasks through the online platform called “Rain Classroom” one week prior to the class. They then engaged in collaborative learning by studying together in groups. Following the instructional video, they completed a group assignment, collected and organized relevant materials, and engaged in group discussions. They addressed questions provided by the instructor, and subsequently, based on the teaching content, posed at least one question while also providing an answer. Finally, they synthesized their findings and prepared a PowerPoint presentation for the subsequent PBL discussion. These pre-course preparations were implemented to facilitate effective pre-class learning.

During the class session, each study group delivered a 5-min presentation using a PowerPoint (PPT), summarizing their completion of the pre-class tasks. The presentation included selecting 1 to 2 questions from those set by the instructor, as well as addressing self-generated questions and providing corresponding answers. While time constraints did not permit every group to report on all topics, a subsequent 3-min discussion period allowed all students in the classroom (with each classroom accommodating up to 4 study groups) to participate in the collective discussion of all topics. The total PBL teaching time ranged from 32 to 35 min per class hour. During this phase, the instructor did not actively participate in the student discussions. However, in the subsequent LBL teaching phase, the instructor highlighted students’ mistakes and identified areas of improvement based on observations made during the pre-class preview (collected through the “Rain Classroom” platform) and subsequent discussions.

Following the completion of the experimental operations, students engaged in group discussions centered around the obtained results, with the instructor providing guidance and intervention as necessary. Post-class, the instructor disseminated additional scholarly articles and online resources through the “Rain Classroom” platform. Students then collaborated in small groups, studying and subsequently writing and submitting their experimental reports.

Throughout the teaching process, students in both the hPBL and LBL groups had the opportunity to engage in online communication with the instructor through the discussion area of the “Rain Classroom” platform, allowing for timely resolution of any learning-related challenges encountered. This mode of communication effectively eliminated the constraints of time and space. Following the completion of all classes, the exam was administered simultaneously.

### Evaluation and analysis

The efficacy of the hPBL teaching approach was evaluated through the analysis of test scores and post-course questionnaires. The assessment of test scores in the medical molecular biology experiment course encompassed four components: experimental theory, experimental operation, experimental report, and practical application. The maximum total score for the test was 100 points, which remained consistent for both the LBL and hPBL groups.

The experimental theory section consisted of 15 multiple-choice questions, each worth two points, resulting in a maximum score of 30 points. The questions assessed students’ understanding of concepts and technical principles.

The experimental operation segment involved 12 microelements, such as DNA extraction, DNA preservation, DNA concentration and purity detection, PCR system establishment, electrophoresis, and result observation. Each microelement was assigned three points, allowing for a maximum score of 36 points in this category.

Students’ performance in the experimental report was evaluated based on their ability to describe the experimental purpose, experimental reagents, experimental methods, and display and analysis of experimental results. Each requirement was worth five points, leading to a maximum score of 20 points for this category.

The practical application section included one experimental design question, in which students were required to design an experiment based on given conditions. The score was assigned based on the rationality, operability, and completeness of the experimental design, with a maximum score of 14 points for this category.

By comparing the scores, comparisons were made between the hPBL and LBL teaching models, as well as between subgroups within the hPBL group (preventive medicine class 2 and medical imaging class 2). Additionally, comparisons were conducted between the two experimental groups consisting of students majoring in preventive medicine.

Upon completion of the course, hPBL students were provided with an anonymous questionnaire to evaluate their learning experiences and perceptions. The questionnaire comprised 10 questions that encompassed various aspects, such as promoting active learning, facilitating systematic mastery of course knowledge, improving the accuracy of experimental operations, enhancing teamwork skills, fostering self-learning ability, improving interpersonal communication and expression skills, enhancing logical thinking and summarization abilities, improving scientific research thinking and innovation abilities, liking hybrid PBL teaching methods, and concerns regarding excessive time consumption.

Students rated each question on a 5-point Likert scale, where 1 indicated strong disagreement and 5 indicated strong agreement. For certain questions (e.g., the question regarding excessive time consumption), the scoring was reversed. In the analysis, agreement (agree and strongly agree) and disagreement (disagree and strongly disagree) were combined. The reliability of the questionnaire was deemed satisfactory, with Cronbach’s alpha coefficient calculated as 0.866.

Data analysis was conducted using SPSS 20.0. An independent sample *T*-test was employed to compare responses between different experimental groups. One-way ANOVA was used to compare responses between different subgroups, while a Chi-square test was utilized to compare rates. A significance level (alpha) of 0.05 was set, and a *P*-value less than 0.05 was considered statistically significant.

## Results

### Comparison of total scores

**FIGURE 2:**
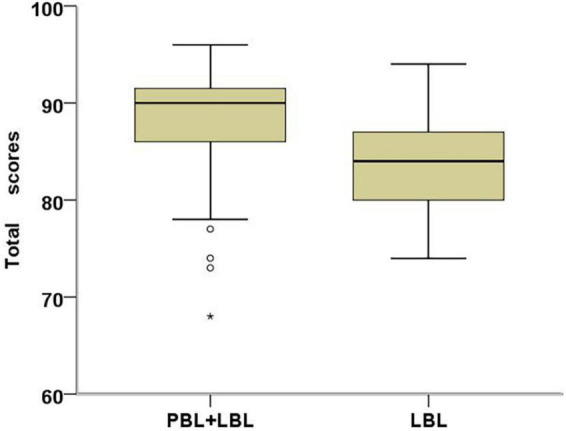
Box plots of total scores in hPBL students (*N* = 83) and LBL students (*N* = 81). Students in the hPBL group scored significantly higher than the LBL group (Cohen’s d = 0.83, *P* = 0.000).

Figure 2 illustrates the overall scores attained by the hPBL (hybrid Problem-Based Learning) group and the LBL (Lecture-Based Learning) group. The total score encompasses performance in the experimental theory test, experimental operation, experimental report, and experimental design. The hPBL group exhibited an average score of 88.22 ± 5.29 points, whereas the LBL group achieved an average score of 84.06 ± 4.77 points. Notably, the hPBL group demonstrated significantly higher total scores compared to the LBL group (*P* < 0.01). It is pertinent to mention that despite four students in the hPBL group obtaining lower grades, two of them even scored below the lowest score observed in the LBL group.

**FIGURE 3:**
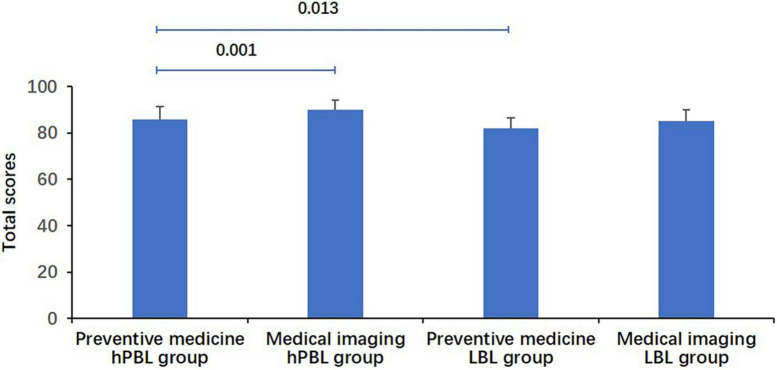
Comparison of total scores for students. The scores of medical imaging students were significantly higher than those of preventive medicine students in the hPBL group (*P* = 0.001). Preventive medicine students in the hPBL group had significantly higher scores than those in the LBL group (*P* = 0.013).

Considering the experimental heterogeneity, a comparison of the total scores between two subgroups within the hPBL group was further conducted (Figure 3). Among students majoring in preventive medicine, the average score was 85.73 ± 5.69 points, while students majoring in medical imaging achieved an average score of 89.86 ± 4.35 points. A one-way ANOVA revealed a statistically significant difference between these two subgroups (*P* = 0.001). Additionally, the total scores of preventive medicine students in the hPBL group were examined and compared with those in the LBL group (Figure 3). The average score for preventive medicine students in the hPBL group was 85.73 ± 5.69 points, whereas their counterparts in the LBL group achieved an average score of 81.89 ± 4.46 points. Remarkably, the total scores of students in the hPBL group remained significantly higher than those of students in the LBL group (*P* = 0.013).

### Analysis of various achievements

The results of individual assessment items were compared and presented in Table 1. Students in the hPBL group demonstrated significantly higher scores in experimental theoretical knowledge, experimental report, and experimental design and practice, as compared to students in the LBL group (*P* < 0.01). However, in terms of experimental manipulation skills, although the LBL group had slightly higher scores than the hPBL group, the difference was not statistically significant (*P* = 0.097).

**TABLE 1:** Analysis of students’ experimental scores.

Experimental scores	hPBL (*n* = 83)	LBL (*n* = 81)	*T*-value	*P*-value*
Experimental theoretical knowledge	26.29 ± 2.28	25.05 ± 2.65	3.219	< 0.01
Experimental operation skills	32.37 ± 2.06	32.88 ± 1.78	1.671	0.097
Experimental report	17.74 ± 1.23	15.97 ± 1.17	9.446	< 0.01
Design and practice	11.81 ± 1.26	10.16 ± 1.40	7.910	< 0.01
Grand average	88.22 ± 5.29	84.06 ± 4.77	5.275	< 0.01

*Based on independent sample *T*-test, *P* < 0.05 was considered significant.

**FIGURE 4:**
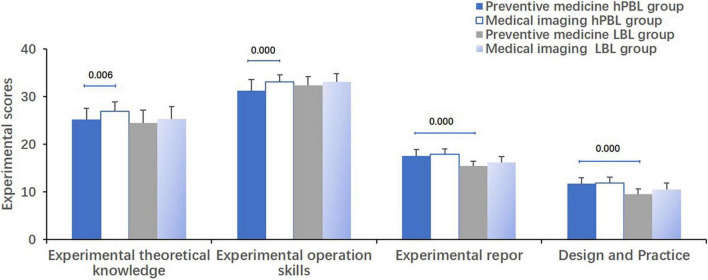
Comparison of experimental scores for students. Comparing the two subgroups within the hPBL group, students majoring in medical imaging exhibited significantly higher scores in experimental theoretical knowledge (*P* = 0.006) and experimental operation skills (*P* = 0.000) compared to students majoring in preventive medicine. Regarding experimental report, design and practice, preventive medicine students in the hPBL group achieved significantly higher scores than those in the LBL group (*P* = 0.000).

When comparing the two subgroups within the hPBL group, students majoring in medical imaging exhibited significantly higher scores in experimental theoretical knowledge (*P* = 0.006) and experimental operation skills (*P* = 0.000) compared to students majoring in preventive medicine. However, there were no significant differences observed in experimental report, design and practice scores between the two subgroups (Figure 4).

Regarding experimental report, design, and practice, preventive medicine students in the hPBL group achieved significantly higher scores than those in the LBL group (*P* = 0.000, Figure 4).

### Questionnaire analysis

In order to gain insights into the learning experience and perceptions of students who participated in the hPBL teaching model, an anonymous questionnaire was administered to the hPBL group students after the course. Participation in the survey was voluntary, and a total of 83 questionnaires were distributed, all of which were completed and returned, resulting in a response rate of 100%. The findings, as presented in Table 2, indicate that students generally held favorable views toward the hPBL method. When asked about the impact of the hPBL model on their systematic mastery of course knowledge, improvement in the accuracy of experimental operations, self-learning abilities, liking of the hPBL teaching method, and the perceived time commitment, the combined percentages of agreement and strong agreement were 88.0, 89.2, 88.0, 89.2, and 24.1%, respectively. However, it is worth noting that there were some differences in opinions among students from different majors (*P* < 0.05, Table 3).

**TABLE 2:** Analysis of students’ views and self-perception ability of the hPBL group.

Survey content	Strongly agree/agree *n* (%)	Neutral *n* (%)	Strongly disagree/disagree *n* (%)
hPBL promoted active learning	76 (91.6)	0	7 (8.4)
hPBL facilitated systematic mastery of course knowledge	73 (88.0)	0	10 (12.0)
hPBL improved the accuracy of experimental operation	74 (89.2)	0	9 (10.8)
hPBL improved teamwork skills	78 (94.0)	0	5 (6.0)
hPBL improved self-learning ability	73 (88.0)	0	10 (12.0)
hPBL improved interpersonal communication and expression ability	78 (94.0)	2 (2.4)	3 (3.6)
hPBL improved logical thinking and summary ability	77 (92.8)	0	6 (7.2)
hPBL improved scientific research thinking and innovation ability	75 (90.4)	1 (1.2)	7 (8.4)
I liked hPBL teaching methods	74 (89.2)	1 (1.2)	8 (9.6)
hPBL consumed too much spare time	20 (24.1)	1 (1.2)	62 (74.7)

**TABLE 3:** Comparative analysis of students’ views and self-perception ability between the two subgroups of hPBL.

Survey content	Preventive medicine *n* (%)	Medical imaging *n* (%)	χ^2^ value	*P*-value*
hPBL promoted active learning	28 (84.8)	48 (96.0)	3.201	0.074
hPBL facilitated systematic mastery of course knowledge	26 (78.8)	47 (94.0)	4.341	0.037
hPBL improved the accuracy of experimental operation	26 (78.8)	48 (96.0)	6.092	0.014
hPBL improved teamwork skills	30 (90.9)	48 (96.0)	0.910	0.340
hPBL improved self-learning ability	26 (78.8)	47 (94.0)	4.341	0.037
hPBL improved interpersonal communication and expression ability	30 (90.9)	48 (96.0)	1.049	0.592
hPBL improved logical thinking and summary ability	31 (93.9)	46 (92.0)	0.111	0.738
hPBL improved scientific research thinking and innovation ability	28 (84.8)	47 (94.0)	2.583	0.275
I liked hPBL teaching methods	26 (78.8)	48 (96.0)	6.324	0.042
hPBL consumed too much spare time	13 (39.4)	7 (14.0)	7.436	0.024

*Based on Chi-square test, *P* < 0.05 was considered significant.

## Discussion

Educators have always been striving to broaden their teaching ideas and improve teaching methods in order to enhance the quality of education. This is particularly important for courses that involve highly theoretical and complex content, such as molecular biology, which can often be perceived as boring and abstract. The hPBL (hybrid Problem-Based Learning) teaching model offers a combination of traditional and innovative teaching methods. It not only facilitates the systematic and precise acquisition of knowledge but also develops students’ ability to flexibly apply that knowledge to analyze and solve problems.

The effectiveness of the hPBL teaching model has been demonstrated to some extent in medical education. However, most of the research has focused on theoretical teaching, with limited application and assessment in the context of experimental courses.

Grades are commonly used as a means to evaluate student performance and the effectiveness of teaching methods, and the hybrid PBL teaching model is no exception. Our research has shown that students in the hPBL group achieved significantly higher scores than those in the LBL group in the medical molecular biology experimental course. These scores reflect students’ performance in acquiring experimental theoretical knowledge, writing experimental reports, and applying the acquired knowledge in practical situations. This indicates that the hPBL teaching model plays a positive role in promoting the teaching of molecular biology experimental courses.

The PBL teaching model is known for its student-centered approach, requiring students to prepare before class, engage in discussions during class, and summarize their learning after class. The integration of PBL teaching into the traditional teaching model has raised concerns among some teachers and students. They worry about whether it would increase the workload for students, affect the systematic and precise mastery of basic knowledge by taking up classroom time, and improve students’ application abilities.

To address these concerns, a further analysis of the evaluation items in the study was conducted. The results revealed that the hPBL group performed significantly better than the LBL group in terms of acquiring experimental theoretical knowledge. This suggests that the integration of the PBL teaching method and the reduction of LBL application time did not compromise the accuracy and systematic acquisition of theoretical knowledge in the study. This finding was also supported by 88% of the students in the questionnaire.

Furthermore, the hPBL group outperformed the LBL group in terms of report writing and practical application. This indicates that the use of hPBL in our study compensated for any deficiencies in the LBL model regarding the integration of theory and practice, and effectively enhanced students’ abilities to analyze and solve problems. In fact, 90.4% of the students affirmed that the hPBL model had improved their innovation abilities. In our study, the hPBL group was given the opportunity to set their own questions and answer them, which may have served as a motivation for students to enhance their analytical and problem-solving skills.

These findings suggest that the hPBL teaching model successfully addresses the concerns raised by the integration of PBL and traditional teaching methods. It not only ensures the acquisition of theoretical knowledge but also enhances students’ practical application skills and innovation abilities.

It is interesting to note that there was no difference in experimental operation skills between the hPBL group and the LBL group. One possible explanation for this could be that the same learning materials were provided to all students through “Rain Classroom” before class, which included short videos demonstrating the experimental procedures. This might have generated more interest among the students and motivated them to dedicate more time to studying, particularly for the LBL students.

Upon analyzing the grades, it was observed that although the overall grades of the hPBL group were significantly higher than those of the LBL group, there were four students in the hPBL group who had lower grades. Additionally, two of these students had even lower grades than the lowest score in the LBL group. Here, it is agreed with Jia et al. (19) that Chinese students are accustomed to and have been receiving LBL education since elementary school. This may be the reason that these few students in the hPBL group struggled to quickly adapt to the addition of the PBL method, resulting in lower grades.

It is important to consider that the transition from a traditional LBL approach to a more student-centered PBL approach can be challenging for some students, particularly if they have been accustomed to a different method of learning for a long time. To address this issue, it may be beneficial to provide additional support and resources to help these students adapt to the PBL teaching method. This could include extra guidance, targeted interventions, or personalized assistance to ensure their successful integration into the hPBL group. While the majority of students benefited from the hPBL model, it is crucial to recognize and address the challenges faced by a small number of students during the transition period. By providing appropriate support, these students can be helped to overcome any difficulties and fully benefit from the advantages of the PBL teaching method.

The hPBL teaching model has shown to promote self-directed learning and enhance students’ self-learning abilities, and it has been well-received by the majority of students. Our research indicates that while both groups of students were provided with the same learning materials prior to class, the hPBL group was tasked with answering teacher-posed questions, whereas the LBL group was not required to complete this task. This requirement for self-study and material exploration, utilizing textbooks, the internet, and library resources to seek satisfactory answers, drove students in the hPBL group to invest more time and effort compared to their counterparts in the LBL group. Consequently, this process stimulated active learning awareness and exercised their self-learning abilities.

Evidence from the “Rain Classroom” records reveals that students in the hPBL group, on average, watched each learning video 2.13 times, whereas students in the LBL group watched only 0.97 times. Active communication between students and teachers was also observed in the “Rain Classroom” message board, where the hPBL group demonstrated more engagement compared to the LBL group. Additionally, students in the hPBL group engaged in group discussions to exchange and summarize collected data, followed by creating PowerPoint presentations for classroom delivery. As a result, 92.8% of students felt that the hPBL model enhanced their summarization skills, while 94.0% believed it improved their cooperation and communication abilities. Such acquired skills are seen as valuable assets for future employment, enabling students to perform better and garner popularity in their chosen careers.

In China, third-year medical students face relatively heavy academic demands. To prevent further burden on students due to the integration of the hPBL teaching model, teachers undertake significant preparation work. They carefully curate and produce learning materials, which are then released to students through the “Rain Classroom” platform. The advantage of video materials is their ability to be stored and replayed indefinitely. This allows students to flexibly manage their time according to their individual circumstances and facilitates timely communication with teachers through the “Rain Classroom” platform. For each independent experiment, teachers set a maximum of seven questions, serving as a guide for students’ independent learning. However, despite these efforts, 24.1% of students still feel that the hPBL approach occupies too much of their time.

It must be recognized that devoting additional study hours to pre-lecture, during-lecture, and post-lecture activities can indeed pose a significant challenge, particularly given the extensive medical syllabus that needs to be covered. Nevertheless, the approach mitigates this challenge. Firstly, the experimental course is prioritized, comprising a limited number of classes, thereby keeping the quantity of pre- and post-class activities manageable. Implementing the hPBL model for experimental teaching can ensure that students are not overwhelmed. Secondly, for other courses, a mixed teaching approach is recognized as valuable, especially when focusing on key topics that demand deeper comprehension. By narrowing the focus to a select few chapters for in-depth exploration and practice, students benefit from a more targeted and profound understanding of these crucial topics. While this may increase the overall workload, the enhanced comprehension it fosters is considered well worth the effort.

Additionally, variations in opinions were observed among students from different majors during the survey. For instance, differences emerged between the two subgroups of the hPBL group regarding the acquisition of experimental theoretical knowledge, practical skills, improvement in self-learning abilities, and overall satisfaction with this teaching format. One possible explanation for these differences is that although both preventive medicine and medical imaging students are admitted through the Chinese college entrance examination, the admission scores for the medical imaging major are higher than those for the preventive medicine major in the school. Moreover, credit scores vary between the two majors in the two years following admission. These disparities suggest that the hPBL teaching method yields different improvement effects based on students’ foundational knowledge and learning abilities, consequently impacting their satisfaction levels. Therefore, when implementing hPBL in the future, it is crucial to adjust the proportion of PBL appropriately based on the characteristics and needs of different student cohorts to achieve the optimal teaching effect.

Indeed, their study does present several limitations. Primarily, the research was exclusively conducted among students majoring in preventive medicine and medical imaging. The teaching efficacy of the hPBL model, as well as the optimal proportion of PBL implementation, require further evaluation for students enrolled in clinical medicine and nursing programs, which have higher and lower admission scores, respectively. Secondly, their assessments were conducted immediately post-course, leaving the long-term effects of hPBL on knowledge retention unexplored. To address this gap, they have planned a longitudinal follow-up study to evaluate the enduring impact of hPBL on knowledge retention and practical application. Thirdly, they acknowledge the possibility of a positive bias among students toward the novelty of the new teaching model, potentially leading them to respond to questions in an idealistic manner. This potential bias should be taken into consideration when interpreting the results. Lastly, the scope of their research was limited to a single course. It would be beneficial to investigate whether the hybrid PBL model can be effectively implemented across other experimental courses within Basic Medicine. This aspect will be incorporated into their follow-up observational study, thereby contributing to a broader adoption and promotion of the hybrid PBL model. These limitations, while offering avenues for further research, should be considered when drawing conclusions from the current study.

## Conclusion

This study utilizes the “Rain Classroom” platform to integrate Problem-Based Learning (PBL) principles into conventional Lecture-Based Learning (LBL) practices. Through an analysis of students’ academic performance and satisfaction levels, it is observed that, at the present stage, the implementation of the hybrid PBL teaching model within medical molecular biology experimental courses for students majoring in preventive medicine and medical imaging at our institution effectively enhances the quality of instruction. This approach notably enhances students’ capacities in self-directed learning, teamwork, communication, analysis, problem-solving, synthesis, and innovation, thereby fostering the development and refinement of students’ overall competencies. The hybrid PBL teaching model emerges as a promising strategy for enhancing academic achievement and experimental proficiencies in the realm of medical higher education.

## Data availability statement

The original contributions presented in this study are included in this article/Supplementary material, further inquiries can be directed to the corresponding author.

## Ethics statement

The studies involving human participants were reviewed and approved by the Ethics Committee of Beihua University. The patients/participants provided their written informed consent to participate in this study.

## Author contributions

MQ: Conceptualization, Data curation, Formal analysis, Funding acquisition, Investigation, Methodology, Project administration, Resources, Supervision, Writing – original draft, Writing – review & editing. QH: Data curation, Project administration, Software, Writing – review & editing. CY: Data curation, Writing – review & editing. XL: Conceptualization, Data curation, Writing – review & editing. JX: Data curation, Writing – review & editing. ZD: Conceptualization, Funding acquisition, Methodology, Resources, Supervision, Writing – review & editing.
