# Supplementary material for: Application and evaluation of the hybrid “Problem-Based Learning” model based on “Rain Classroom” in experimental courses of medical molecular biology
Source: Front Med (Lausanne). 2024 Jul 25;11:1334919. doi: 10.3389/fmed.2024.1334919 (PMC11306937; doi:10.3389/fmed.2024.1334919)
Supplement: Supplementary file 1 [file Data_Sheet_1.PDF]

ATTACHED TABLE | Class hour arrangement of molecular biology experiment and hPBL pre-class thinking questions.

Schedule	Specific operation	Thinking questions
Genomic DNA extraction from eukaryotic cells<br><br>300 min	1. Preparation of isolated rabbit leukocytes<br>2. Extraction of genomic DNA<br>3. Purification of DNA extracts<br>4. Preservation of DNA in TE solution	1. Characteristics of eukaryotic genomes.<br>2. Extraction principle of genomic DNA.<br>3. What was the role of the reagent protease K?<br>4. What was the mechanism by which ethanol precipitates DNA?<br>5. What were the differences in methods between genomic DNA extraction and plasmid DNA extraction?<br>6. Which instruments were used?<br>7. Asked at least one question and answered it.
Identification of nucleic acids<br><br>60 min	1. Preparation of DNA test samples<br>2. Detection of DNA concentration and purity	1. Principle of determining nucleic acid concentration by ultraviolet spectrophotometry.<br>2. Principle of determining the purity of nucleic acid by ultraviolet spectrophotometry.<br>3. What was the basis for setting the concentration of fluorescent standard?<br>4. What were the standards for the concentration and purity of DNA extracts to meet the requirements of PCR amplification?<br>5. Asked at least one question and answered it.
PCR amplification of genomic DNA fragments<br><br>180 min	1. Establishment of amplification system<br>2. Amplification of GAPDH gene fragment, 428bp in length	1. Basic principle of PCR amplification.<br>2. Composition of the PCR system.<br>3. How to design primers.<br>4. How to determine the annealing temperature?<br>5. Why were the upstream and downstream primers respectively added into the PCR reaction system?<br>6. What determined the number of cycles?<br>7. Asked at least one question and answered it.
Agarose gel electrophoresis of DNA	1. Gel preparation<br>2. Add the samples<br>3. Electrophoresis	1.Principle of agarose gel electrophoresis.<br>2. What were the commonly used

180 min	4. Observation of the results	nucleic acid stains?<br>3. What was the principle of EB dyeing?<br>4. What were the commonly used agarose gel electrophoresis buffers?<br>5. What were the similarities and differences between DNA detection by polyacrylamide gel electrophoresis and DNA detection by agarose gel electrophoresis?<br>6. Asked at least one question and answered it.
